# Melatonin Protects Bovine Spermatozoa by Reinforcing Their Antioxidant Defenses

**DOI:** 10.3390/ani13203219

**Published:** 2023-10-15

**Authors:** Sophia N. Lavrentiadou, Vasiliki Sapanidou, Elena E. Tzekaki, Ioannis Margaritis, Maria P. Tsantarliotou

**Affiliations:** 1 Laboratory of Physiology, Department of Animal Structure and Function, School of Veterinary Medicine, Faculty of Health Sciences, Aristotle University of Thessaloniki, 54124 Thessaloniki, Greece; vasilikisap@windowslive.com (V.S.); johnmarvet@yahoo.gr (I.M.); mtsant@vet.auth.gr (M.P.T.); 2 Laboratory of Biochemistry, Department of Chemistry, Aristotle University of Thessaloniki, University Campus, 54124 Thessaloniki, Greece; etzekaki@chem.auth.gr

**Keywords:** antioxidant capacity, glutathione, hydrogen peroxide, nitric oxide synthase, oxidative stress, superoxide ion, sperm physiology, reactive oxygen species

## Abstract

**Simple Summary:**

In bovine reproduction, genetically superior semen is customarily frozen to ensure its conservation and broad use. Freezing/thawing and handling compromise the quality and fertilizing capacity of spermatozoa, mainly because they induce oxidative stress, an imbalance between reactive oxygen or nitrogen species and antioxidants. The first are highly reactive molecules that alter almost all cellular components (proteins, lipids, DNA) and damage cells. Antioxidants, on the other hand, neutralize these reactive species, thus protecting cells and their components. Supplementation of media with antioxidants has been tested to reinforce the antioxidant defenses of spermatozoa and prevent oxidative stress. In this study, the antioxidant/protective role of the hormone melatonin on post-thawing spermatozoa was assessed. The inclusion of melatonin in the media, even after exposure of spermatozoa to hydrogen peroxide, enhanced the antioxidant status, preserved the semen characteristics and potentially the fertilization capacity of bovine spermatozoa. We anticipate that these results will contribute to the optimization of sperm handling protocols in ART.

**Abstract:**

Cryopreserved semen is widely used in assisted reproductive techniques. Post-thawing spermatozoa endure oxidative stress due to the high levels of reactive oxygen and nitrogen species, which are produced during the freezing/thawing process, and the depletion of antioxidants. To counteract this depletion, supplementation of sperm preparation medium with antioxidants has been widely applied. Melatonin is a hormone with diverse biological roles and a potent antioxidant, with an ameliorative effect on spermatozoa. In the present study, we assessed the effect of melatonin on thawed bovine spermatozoa during their handling. Cryopreserved bovine spermatozoa were thawed and incubated for 60 min in the presence or absence of 100 μΜ melatonin. Also, the effect of melatonin was assessed on spermatozoa further challenged by the addition of 100 μΜ hydrogen peroxide. Spermatozoa were evaluated in terms of kinematic parameters (CASA), viability (trypan blue staining) and antioxidant capacity (glutathione and NBT assay, determination of iNOS levels by Western blot analysis). In the presence of melatonin, spermatozoa presented better kinematic parameters, as the percentage of motile and rapid spermatozoa was higher in the melatonin group. They also presented higher viability and antioxidant status, as determined by the increased cellular glutathione levels and the decreased iNOS protein levels.

## 1. Introduction

The use of cryopreserved semen is widely applied in assisted reproductive techniques (ART) for the apparent reason that it enables broad use and conservation of genetically superior semen. Undoubtedly, the quality of semen post thawing determines the success of all processes applied in ART [1]. Despite the advances in cryopreservation of bull semen, the viability of spermatozoa after thawing is still relatively low and highly variable [2], mainly because of an imbalance in the redox status of spermatozoa. Freezing induces alterations in membrane fluidity, which damage mitochondrial membranes, disrupt mitochondrial membrane potential and produce excessive amounts of reactive oxygen species (ROS) [3,4]. The high content of polyunsaturated fatty acids (PUFAs) in the cell membranes of spermatozoa render them particularly vulnerable to oxidation by ROS [5]. Moreover, during their preparation for ART, spermatozoa are deprived of the antioxidants that are present in the seminal plasma and are exposed, during their handling, to oxygen, temperature changes and mechanical stress (e.g., shearing forces), which increase the production of ROS. Thus, the intracellular antioxidant defenses of spermatozoa are depleted by the overload of ROS produced during their preparation for ART and the cells endure oxidative stress (OS), manifested as lipid peroxidation (LPO), DNA damage and apoptosis [6,7,8]. To prevent this, researchers have employed supplementation of antioxidants in the sperm preparation media to preserve the fertilizing ability of spermatozoa during these processes and to improve male reproductive performance [9,10,11,12,13]. The role of antioxidants in male reproductive physiology has been investigated in vivo, through oral supplementation, or in vitro, by supplementation of culture media [12,14]. Currently, the consensus is that the inclusion of antioxidants in the sperm preparation media may significantly improve the quality parameters of spermatozoa (for a review, see [10]).

Hydrogen peroxide has been extensively used to study the effects of ROS on sperm physiology. Low concentrations of hydrogen peroxide, typically ranging from 10 μM to 100 μM, are often used to study the physiological effects of ROS. Hydrogen peroxide at these concentrations is considered to mimic the effect of ROS that are naturally present in the reproductive tract. Higher concentrations of hydrogen peroxide, ranging from 100 μM to 1 mM or even higher (up to 5 mM), are commonly used to induce OS in spermatozoa and investigate the detrimental effects of oxidative damage or assess the protective effects of antioxidants [15,16].

Melatonin is an amphiphilic hormone produced in several tissues but mainly in the pineal gland [17]. In addition to its apparently diverse biological roles, such as regulation of the circadian cycle, immune response, and seasonal reproduction [18], melatonin is also a potent antioxidant [19]. Its antioxidant properties are manifested either directly by neutralization of free radicals or indirectly by inducing the activity and expression of key antioxidant enzymes, such as glutathione peroxidase, catalase, and superoxide dismutase (for a review, see [17]). Melatonin is also present in seminal plasma [20]. The ameliorative effect of melatonin as an additive in sperm preparation media has been assessed in many species, such as human [21,22], red deer [23], sheep [24], swine [25] and cattle [26,27,28,29]. In addition to scavenging ROS, melatonin may also exert its biological effect through its receptors MT1 and MT2, which are classic G protein-coupled receptors (GPCRs) that stimulate downstream signaling cascades by activating G proteins [22,30,31]. It appears that melatonin may mediate its biological effects in a dose-dependent manner [32,33,34]. To date, the consensus is that at micromolar concentrations, melatonin reduces ROS and cAMP levels, thus modulating sperm capacitation, acrosome reaction and fertilization rate [23,29,35,36]. At nanomolar concentrations, melatonin interacts with intracellular ligands and modulates the activity of antioxidant enzymes, whereas at picomolar concentrations it binds to cell membrane receptors, thus triggering downstream signaling pathways [37,38]. Melatonin action has also been correlated with the production of nitric oxide (NO) and the expression of the inducible isoform of nitric oxide synthase (iNOS) in macrophages and other cell types [39]. The NO synthases (NOSs) comprise a family of enzymes which utilize oxygen and the amino acid L-arginine to produce the highly reactive molecule NO [40]. Nitric oxide and the nitrogen species which are derived from NO mediate important roles in reproductive processes.

The addition of natural or synthetic antioxidants in the medium of spermatozoa is an effective approach to preventing OS and thus counteract the negative effects of ROS on spermatozoa during ART [41,42,43]. We have previously shown the ameliorative effects of crocin and crocetin on post-thawing spermatozoa [11,13,44], while melatonin has a proven biological role in reproductive processes by acting either as an antioxidant or as a ligand for the melatonin receptors [18,20,30]. The aim of the present study was to investigate if supplementation of melatonin in the sperm preparation media can preserve the quality characteristics of spermatozoa over time after thawing. These findings will set the grounds for the use of melatonin in AI or IVEP protocols to protect spermatozoa from oxidative attack.

## 2. Materials and Methods

### 2.1. Reagents

All reagents were purchased from Sigma Aldrich Co. (Darmstadt, Germany), unless otherwise specified.

Sperm TALP (Tyrode’s albumin lactate pyruvate) containing 100 mM NaCl, 3.1 mM KCl, 25 mM NaHCO_3_, 0.29 mM NaH_2_PO_4_, 21.6 mM Na lactate, 2 mM CaCl_2_, 1.5 mM MgCl_2_, 10 mM Hepes, 1 mM sodium pyruvate and 50 μg/mL gentamycin in water for embryo transfer [45] was equilibrated overnight at 37 C in a 5% CO_2_ humidified atmosphere before use.

A fresh stock solution of 10 mM melatonin (Cayman, Clinical, Ann Arbor, MI, USA) was prepared before each experiment. Melatonin was diluted in dimethylosulfoxide (DMSO) and was added to the samples to a final concentration of 100 μM. DMSO (vehicle) was added in all melatonin-free samples to a final concentration of 1% (*v*/*v*), similar to that in the melatonin samples.

RIPA buffer consisted of 50 mM Tris-HCl, pH 7.2, 150 mM NaCl, 1% NP-40, 1 mM EDTA, 1 mM EGTA and 1% (*v*/*v*) of protease inhibitor cocktail (Sigma-Aldrich, Darmstadt, Germany).

The anti-iNOS rabbit polyclonal antibody (18985-1-AP) and the alkaline phosphatase-conjugated anti-rabbit IgG goat monoclonal antibody (SA00002-2) were purchased from Proteintech Group (Manchester, UK).

### 2.2. Semen Collection, Sample Preparation and Experimental Design

Six mature, healthy bulls (three Simmental, two Holstein and one Brown Swiss) of proven fertility (housed at the Department of Artificial Insemination, Directorate of Veterinary Centre of Thessaloniki, National Ministry of Rural Development and Food, Ionia, Greece) were used as sperm donors throughout the study. The age of the animals ranged from 48 to 60 months at the beginning of the experiment. Four different ejaculates were collected from each bull with the use of an artificial vagina. Samples were collected from all animals during the period November 2022–February 2023. Ejaculates were evaluated by the same person; only semen characterized with >70% initial motility, >75% viability and a concentration of at least 600 × 10^6^ spermatozoa/mL of ejaculate was cryopreserved. Semen was diluted in a Tris-citric acid home-made extender containing glycerol and egg yolk (20% Tris-egg yolk, 7% glycerol, 78 mM citric acid, 69 mM fructose, 50 μg tylosin, 250 μg gentamycin, 150 μg lincomycin, 300 μg spectinomycin in each ml of extended frozen semen) and packed into 0.5 mL plastic straws with 50 × 10^6^ spermatozoa/mL. The straws were labeled with the name and registration code of the bull and the date of freezing, then they were allowed to equilibrate at 5 °C for 4 h, after which they were placed on a horizontal rack, 3 cm above the surface of liquid nitrogen, in order to be bathed in liquid nitrogen vapors and thus reach −100 °C within 7–10 min. Once this temperature was reached, the straws were plunged and stored in liquid nitrogen (−196 °C) for 2–4 months.

**Figure 1:**
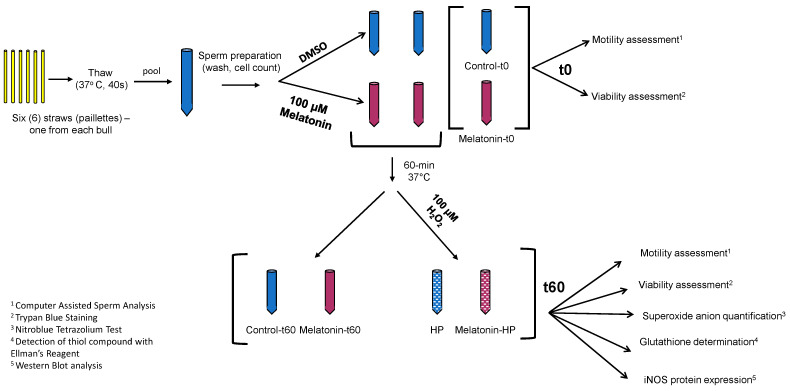
Schematic diagram of the experimental procedure. In each experiment, 6 straws, one from each bull, were thawed, pooled and washed. The suspension of spermatozoa was divided into 6 samples: 3 received 10 μL of 10 mM melatonin solution in DMSO (red tubes), and 3 received equal volume of DMSO (blue tubes). Immediately (t0), two samples (Control-t0, Melatonin-t0) were analyzed by CASA for determination of motility, and microscopically for determination of viability. The other 4 were incubated at 37 °C for 60 min, at which point 100 μM H_2_O_2_ (HP) was added in one control (blue) and in one melatonin (red) tube. All 4 samples (Control-t60, Melatonin-t60, HP and Melatonin-HP) were analyzed with all assays indicated.

One straw (from a single ejaculate) from each bull was thawed by immersion in a 37 °C water bath for 40 s for each experiment. The six samples (one from each bull) were pooled together into a sterile plastic tube (CellstarTubes, Greiner Bio One, Frickenhausen, Germany), washed twice with 3 × volumes of Sperm TALP and centrifuged at 200× *g* for 10 min at room temperature (RT). Sperm concentration was determined by cell count on a Neubauer chamber (OptikLabor, Grale HDS, New South Wales, Australia). Finally, cells were re-suspended in Sperm TALP to a final concentration of 50 × 10^6^ spermatozoa/mL and the sample was divided into six groups (Figure 1), with a total volume of 100 μL each. In four groups, only DMSO (vehicle) was added, while the other two were supplemented with 100 μM melatonin. One DMSO sample (Control-t0) and one melatonin sample (Melatonin-t0) were analyzed immediately. The other four samples were incubated in a 37 °C water bath for 60 min. At the end of the incubation (t60), 100 μΜ H_2_O_2_ was added to one of the DMSO groups (HP) and to one of the melatonin groups (Melatonin-HP) and all four groups were analyzed as described below. Therefore, in each experiment there were 6 samples to be analyzed: Control-t0, Control-t60, HP, Melatonin-t0, Melatonin-t60 and Melatonin-HP, where HP is hydrogen peroxide (100 μM H_2_O_2_). A total of six samples were analyzed in every experiment.

### 2.3. Motility Assessment

Motility assessment was performed by a Computer Assisted Sperm Analyzer (CASA) using Integrated Semen Analysis System Software (V1.2), ISAS MvCo, Valencia, Spain). The CASA system consisted of a triocular optical phase microscope (Nikon Eclipse C1, Nikon, Tokyo, Japan), equipped with a heated plate (Tokai, Tokyo, Japan) and a Baler Scout CCD digital camera (Basler Vision Technologies, Ahrensburg, Germany), connected to a computer. Each time, a 5 μL aliquot of sperm suspension was placed on a pre-warmed slide on the heated plate at 37 °C and 9 different parameters were measured: spermatozoa with rapid, medium, and slow movement, static spermatozoa, progressive motility (PM), curvilinear velocity (VCL), straight line velocity (VSL), average path velocity (VAP) and amplitude lateral head (ALH). CASA default settings: 60 frames/second, total of 25 frames captured; minimum contrast of 80 and medium cell size of 5 pixels; a cutoff value for progressive cells of 50 μm/s for VAP and 70% for medium threshold straightness; static cells: VAP cutoff 25 μm/s and VSL cutoff 10 μm/s.

### 2.4. Viability Assessment

At the indicated time points (0, 60 min), 10 μL of each sample were mixed with an equal volume of trypan blue solution and cells were smeared on a slide [46]. Two hundred spermatozoa in each slide were evaluated microscopically (×1000) as alive (unstained) or dead (stained).

### 2.5. Preparation of Whole Cell Lysates

To measure intracellular content, spermatozoa were resuspended in RIPA buffer and subjected to three cycles of freezing (30 min at −20 °C)–thawing (30 min on ice) followed by three cycles of sonication at 28 kHz for 30 sec. Whole-cell lysates were centrifuged at 10,000× *g* for 10 min to remove debris and the supernatant was used for further analysis.

### 2.6. Determination of Intracellular Glutathione (GSH)

To determine intracellular reduced glutathione (GSH) levels, spermatozoa lysates were incubated with 0.33 mM DTNB [5,5′-dithiobis (2-nitrobenzoic acid)] [47]. The thiol groups of GSH in the lysates cleave the disulfide bond in DTNB to yield 2-nitro-5-thiobenzoic acid, which ionizes to the TNB^2−^ dianion that has a yellow color and can be quantified at 412 nm using a spectrophotometer (Pharmacia LKB-Novaspec II, Northwich, Cheshire, UK). Results were expressed as percentage (%) of control.

### 2.7. Determination of the Intracellular Superoxide Anion Content

The nitroblue–tetrazolium (NBT) assay was performed to evaluate the intracellular generation of the superoxide anion (O_2_^−^) [48,49]. The NBT test is based on the production of blue-purple NBT formazan crystals, as a result of the reduction of yellow NBT chloride [2,2′-bis(4-nitrophenyl)-5,5′-diphenyl-3,3′-(3,3′-dimethoxy-4,4′diphenylene) ditetrazolium chloride] by O_2_^−^. The NBT was dissolved in Dulbecco’s PBS at 5 mg/mL and administered to the whole-cell suspension. Spermatozoa were incubated with NBT (final concentration 0.5 mg/mL) for 60 min and subsequently were washed twice with PBS and centrifuged (300× *g*) for 10 min. Cells and the formed formazan crystals were dissolved in 120 μL of 2 M ΝaOH and 140 μL of DMSO. Optical density was assessed at 630 nm with a ΒioTek EL800 microplate reader (Thomas Scientific, Swedesboro, NJ, USA).

### 2.8. Western Blot Analysis

The intracellular levels of inducible nitric oxide synthase (iNOS) were determined by Western blotting as previously described [50]. A total of 50 μg of total protein were analyzed on a 10% SDS polyacrylamide gel and then transferred onto nitrocellulose membrane. iNOS protein was detected with the iNOS antibody (1:1000) and a secondary alkaline phosphatase-conjugated antibody (1:2000). Nitroblue tetrazolium (NBT), 5-bromo-4-chloro-3- indolyl phosphate (BCIP) and p-nitrophenyl phosphate were used for the alkaline phosphatase reaction. iNOS protein quantitation was performed by densitometry of the appropriate molecular weight band using the ImageJ 1.53a software. The analysis was performed 4 times with samples from independent experiments.

### 2.9. Statistical Analysis

Statistical tests exploited in the study were chosen after performing analyses for the normality of residuals. Non-parametric tests were employed when values did not follow the Gaussian distribution when verified by the normality tests run (Anderson–Darling, D’Agostino-Pearson omnibus, Shapiro–Wilk and Kolmogorov–Smirnov tests). A standard one-way ANOVA was performed for the statistical examination of rapid motility, viability and NBT test. The Kruskal–Wallis test, followed by Dunn’s multiple comparison test, was used for progressive motility, total motility and viability. GSH data were processed with the Kruskal–Wallis test and pairwise comparisons were performed with the Mann–Whitney U test. 

The statistical significance level was set at *p* ≤ 0.05. All experimental data were analyzed with the SPSS version 25.0 (IBM Corp., Armonk, NY, USA) and are presented as mean ± SEM (standard error of the mean).

## 3. Results

### 3.1. Melatonin Inhibits the Loss of Motility over Time during the Handling of Spermatozoa 

**Figure 2:**
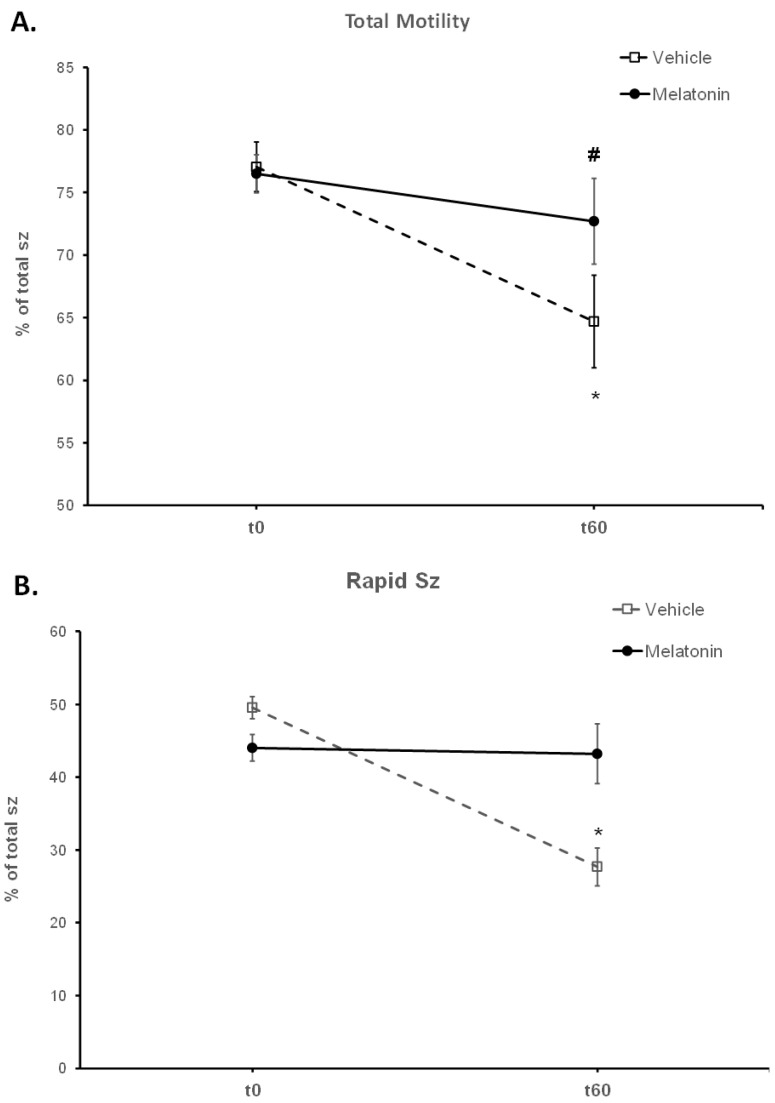
The effect of melatonin on the motility of spermatozoa (sz). Frozen–thawed spermatozoa were incubated at 37 °C for 60 min in the presence of DMSO (Vehicle) or 100 μM melatonin (Melatonin). The percentage of motile spermatozoa (**A**) and the percentage of spermatozoa with rapid motility (**B**) were determined by CASA and the results are presented as mean values (% of total spermatozoa) ± SEM. The asterisk (*) indicates statistically significant difference between Control-t0 and Control-t60 (*p* < 0.01, *n* = 6), whereas the hashtag (#) indicates the tendency of melatonin to increase the percentage of total motile spermatozoa compared to the control group, at t60.

Frozen–thawed spermatozoa undergo OS-induced damage during their preparation for ART. In this experimental system, incubation of bovine spermatozoa at 37 °C for 60 min negatively affected their motility parameters (Table 1). The percentage of motile spermatozoa was reduced during the incubation period from 77.05% at t0 to 64.68% at t60 (*p* = 0.0016) (Figure 2A). Rapidly moving spermatozoa were reduced from 49.57% at t0 to 27.67% (*p* < 0.0001) at t60 (Figure 2B). Although progressive motility was not affected, VCL (*p* = 0.0053) and ALH (*p* = 0.0152) were also reduced during the 60 min incubation (Table 1).

Spermatozoa treated with melatonin retained their motility during the 60 min, as no change was observed in any of their motility parameters between t0 (Melatonin-t0) and t60 (Melatonin-t60) (Figure 2, Table 1). After 60 min of incubation, melatonin retained a higher percentage (43.22%) of rapid spermatozoa (*p* = 0.0002) compared to the Control-t60 group (27.67%) (Figure 2B). Also, there was a tendency (*p* = 0.0655) for a higher percentage (72.68%) of motile spermatozoa in the Melatonin-t60 group (Figure 2A). Moreover, VCL (*p* = 0.007), VAP (*p* = 0.0494) and ALH (*p* = 0.01) were higher in the Melatonin-t60 group compared to the Control-t60 group (Table 1).

### 3.2. In the Presence of Melatonin, Spermatozoa Retain Their Motility during Insult by Hydrogen Peroxide

**Figure 3:**
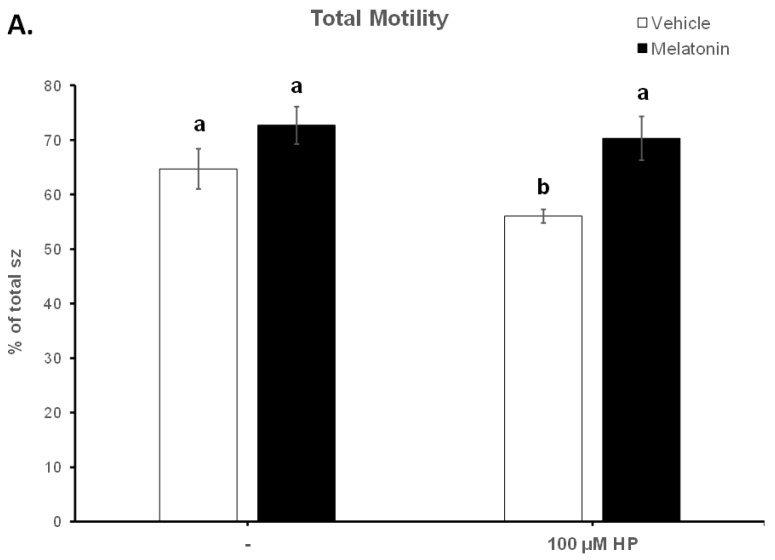

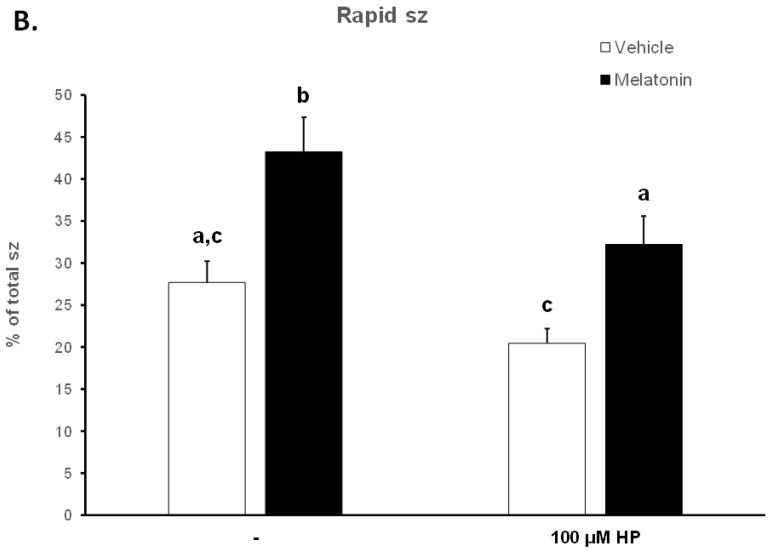
The effect of melatonin on the percentage of total motility (**A**) and rapid spermatozoa (**B**) after exposure to 100 μΜ H_2_O_2_. Cells were incubated at 37 °C in the presence of DMSO (vehicle, white bars) or 100 μΜ melatonin (black bars) for 60 min, at which point the indicated samples were exposed to 100 μΜ hydrogen peroxide (HP). The percentages of spermatozoa (sz) with total (**A**) or rapid (**B**) motility were determined by CASA. Results are presented as mean values (% of total spermatozoa) ± SEM and different letters indicate statistically significant differences (*p* < 0.05, *n* = 6).

The deterioration of spermatozoa over time during their handling has been attributed to the generation of ROS [51]. To augment this effect, 100 μM H_2_O_2_ was added to the medium by the end of the 60 min incubation period. As anticipated, hydrogen peroxide (HP) had an overall negative effect on the motility of spermatozoa and mediated a further reduction of the motility parameters at t60 (Figure 3, Table 1). In particular, hydrogen peroxide induced an additional reduction in total motility from 64.68% in the Control-t60 group to 56.03% (*p* = 0.0471) in the HP group. Melatonin alleviated this effect and retained the percentage of motile spermatozoa at levels (70.32%) higher than the HP group (*p* = 0.0015) and similar to those of the Melatonin-t60 group (72.68%) (Figure 3A). Also, regardless of the presence of hydrogen peroxide, melatonin retained the percentage of rapid spermatozoa at levels that were similar to the percentage of Control-t0 group and significantly higher than those (20.48%, *p* = 0.0043) of the HP group (Figure 3B). Melatonin had similar effect on other kinematic parameters, as it retained higher VCL, VAP and ALH values compared to the H_2_O_2_-treated cells (Table 1). These data demonstrate that pre-incubation of spermatozoa with melatonin preserves their movement characteristics even under conditions of acute increase of hydrogen peroxide.

### 3.3. Melatonin Protects Viability of Spermatozoa against Oxidative Stress

**Figure 4:**
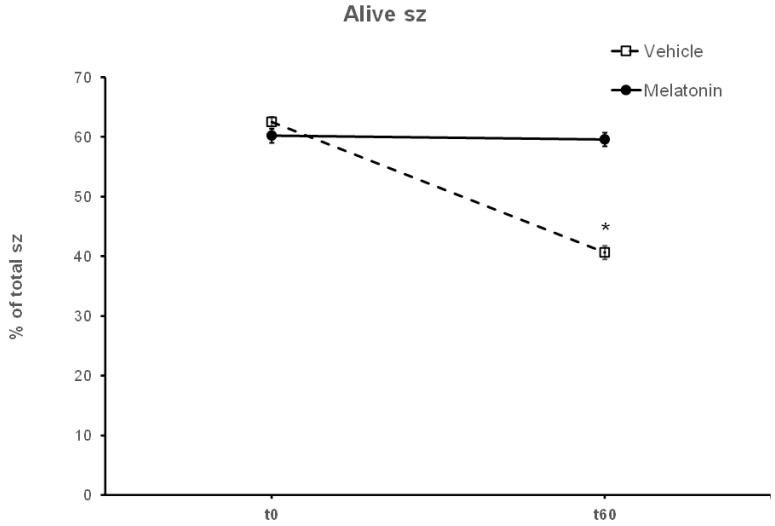
The effect of melatonin on the viability of spermatozoa (sz). Frozen–thawed spermatozoa were incubated at 37 °C for 60 min in the presence of DMSO (Vehicle) or 100 μM melatonin (Melatonin). The percentage of live spermatozoa was determined by optical microscopy after staining of spermatozoa with trypan blue solution. The results are presented as mean values (% of total spermatozoa) ± SEM (*n* = 6). The asterisk (*) indicates statistically significant different values (*p* ≤ 0.01).

**Figure 5:**
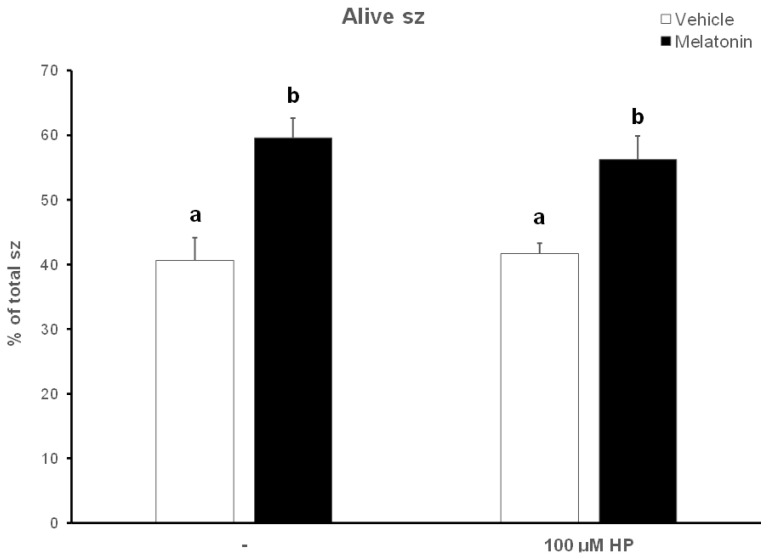
The effect of melatonin on the viability of spermatozoa (sz) after exposure to 100 μΜ H_2_O_2_. Cells were incubated at 37 °C in the presence of DMSO (vehicle, white bars) or 100 μΜ melatonin (black bars) for 60 min, at which point the indicated samples were exposed to hydrogen peroxide (HP), washed and stained with trypan blue solution. Spermatozoa were evaluated as alive (unstained) or dead (stained) by observation on an optical microscope. Results are presented as mean values ± SEM (*n* = 6). Different letters indicate statistically significant differences (*p* < 0.01).

After thawing, 62.5% of the spermatozoa were alive, but during the 60—min incubation at 37 °C this percentage was reduced to 40.63% (*p* < 0.001) (Figure 4). Supplementation with melatonin effectively inhibited this reduction in viability, increasing the percentage of live spermatozoa both in the presence (56.22%, *p* = 0.0107) or absence (59.56%, *p* = 0.0018) of hydrogen peroxide (Figure 4 and Figure 5). The insult by hydrogen peroxide at t60 had no effect on viability compared to the untreated cells, but in any case, both melatonin-treated groups had a higher percentage of alive cells (*p* < 0.01) compared to the control spermatozoa treated with hydrogen peroxide (Figure 5).

### 3.4. In the Presence of Melatonin, Spermatozoa Retain Their GSH Content

**Figure 6:**
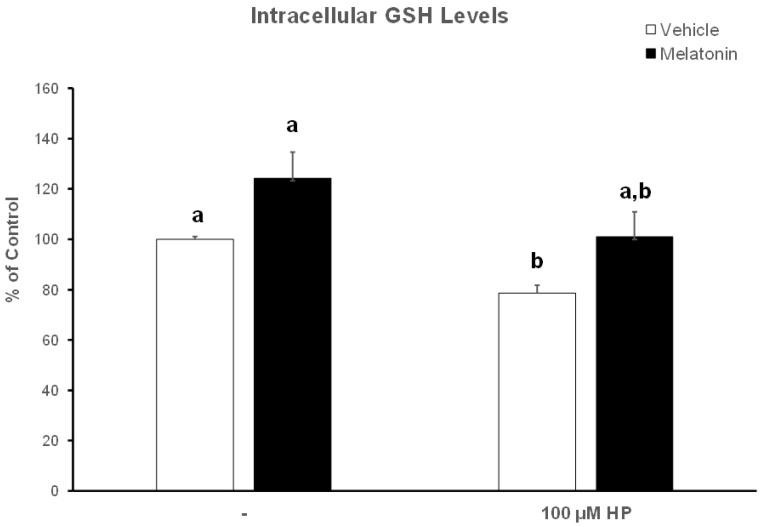
The effect of melatonin on intracellular GSH levels after exposure to 100 μΜ H_2_O_2_. Frozen–thawed bovine spermatozoa were incubated in the presence of DMSO (vehicle, white bars) or 100 μΜ melatonin (black bars) for 60 min, at which point the indicated samples were exposed to hydrogen peroxide (HP). Spermatozoa were lysed and GSH levels in the lysates were determined spectrophotometrically. Results are presented as mean values ± SEM (*n* = 6) and the different letters indicate statistically significant differences (*p* < 0.05).

Hydrogen peroxide depleted spermatozoa of their GSH reserves, as determined by the colorimetric GSH assay and demonstrated in Figure 6. In particular, spermatozoa in the HP group had 22% lower GSH levels compared to the Control-t60 group (*p* = 0.007). In the presence of melatonin, spermatozoa exhibited a tendency to increase intracellular GSH levels to 124.3% of Control-t60. Even after exposure to hydrogen peroxide, the same tendency was observed, with the average GSH levels reaching 101%, as opposed to the 78.5% in the HP group (*p* = 0.063). No statistically significant differences were observed between the Melatonin-t60 and the Melatonin-HP groups.

### 3.5. Melatonin Does Not Affect the Intracellular Levels of Superoxide Ion

**Figure 7:**
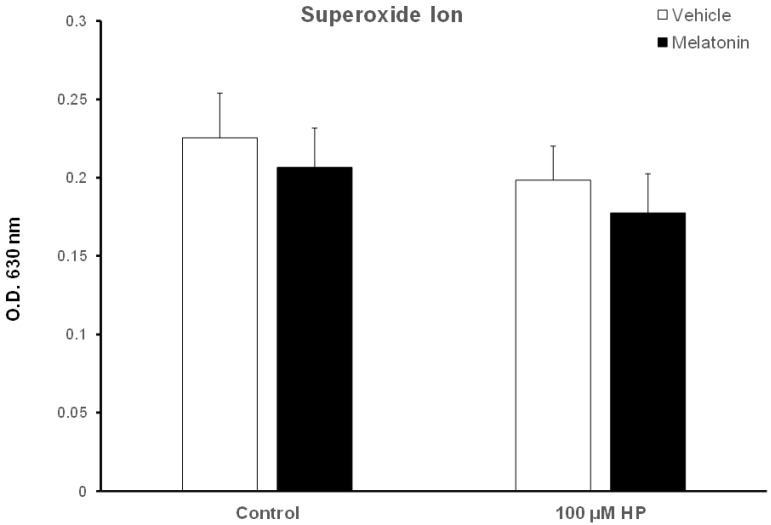
Cellular O_2_^−^ levels in bovine spermatozoa incubated in the presence of DMSO (vehicle, white bars) or 100 μΜ melatonin (black bars) for 60 min, at which point 100 μΜ hydrogen peroxide (HP) was added to the respective groups. Cells were washed and O_2_^−^ levels were determined with the nitroblue-tetrazolium (NBT) assay. Data are presented as mean ± SEM (*n* = 6).

Because superoxide ion (O_2_^−^) is the major ROS of human spermatozoa [52], we determined the effect of hydrogen peroxide and melatonin on O_2_^−^ levels in this experimental system. Surprisingly, exposure of spermatozoa that were incubated for 60 min at 37 °C to 100 μM H_2_O_2_ slightly decreased O_2_^−^ levels (*p* > 0.1) compared to the Control-t60 group (Figure 7, white bars). Exposure of spermatozoa to melatonin for 60 min only slightly reduced intracellular O_2_^−^, although there is no statistically significant difference among these groups (Figure 7, black bars).

### 3.6. Melatonin Enables Spermatozoa to Retain Low Levels of the Inducible Nitric Oxide Synthase (iNOS) 

**Figure 8:**
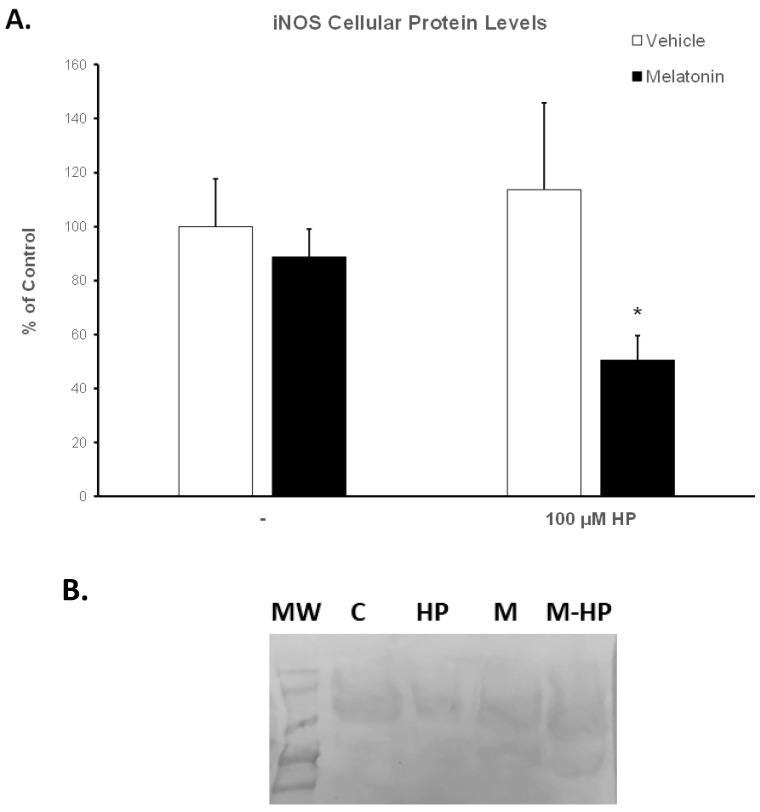
(**A**). Protein levels of iNOS in bovine spermatozoa incubated for 60 min with DMSO (vehicle, white bars) or 100 μΜ melatonin (black bars) and exposed to 100 μΜ H_2_O_2_ (HP), as determined by Western blot analysis. Results are expressed as percentage of the Control-t60. Each bar represents mean values ± SEM (*n* = 4). The asterisk (*) indicates statistically significant differences from the Control-t60 group (*p* < 0.05). (**B**). Representative membrane after blotting with the anti-iNOS antibody (see also Figure S1). MW: molecular weight markers, C: Control-t60, HP: 100 μM H_2_O_2_, M: Melatonin-t60, M-HP: Melatonin-HP.

The expression of the inducible isoform of NOS (iNOS) was determined by Western blot analysis. Figure S1 and Figure 8B show a representative membrane. Hydrogen peroxide had no effect on iNOS levels (Figure 8A, white bars). Spermatozoa incubated for 60 min at 37 °C in the presence of 100 μM melatonin exhibited slightly reduced iNOS levels (88.71%) compared to the Control-t60 group (arbitrarily set at 100%), but this effect was not statistically significant. Interestingly, the addition of hydrogen peroxide in the melatonin-treated spermatozoa further decreased iNOS levels to 50.53% (*p* = 0.0489) of control values (Figure 8A, black bars).

## 4. Discussion

Crucial reproductive processes, both in males and females, are affected by ROS. Regarding spermatozoa, low and moderate concentrations of ROS are required for processes such as capacitation and acrosome reaction [53,54,55]. On the other hand, elevated amounts of ROS reduce sperm motility [56,57]. The excessive amounts of ROS produced during the preparation of spermatozoa for ART are considered a major contributor to the relatively poor outcome of these processes, despite the developments achieved in ART [56,58]. In bovines, the blastocyst rate that is achieved during IVF rarely exceeds 40–50% [59], which is similar to that obtained in previous decades [60]. In the past decade, various antioxidants have been tested as supplements in bovine ART to improve the different steps of these processes, but their effects are still under discussion [9,10,61,62,63,64,65].

Melatonin is a ubiquitously expressed hormone with well-documented antioxidant and free radical scavenging activities [66,67]. However, its exact molecular mechanism(s) of action is (are) yet to be described. Nevertheless, melatonin plays a rather important role in the physiology of reproduction [68,69,70,71] and has been considered as a supplement for media in ART [10,63,72]. In the present study, we assessed the effect of melatonin on frozen/thawed bovine spermatozoa during their incubation at 37 °C and after their challenge with hydrogen peroxide. We also attempted to shed some light on the mechanisms by which melatonin elicits its action. The concentration of hydrogen peroxide was determined based on previous data [22,73,74,75] and pilot experiments. Griveau et al. have demonstrated that 50 μΜ H_2_O_2_ is required to induce capacitation of bovine spermatozoa [73], so a higher concentration was chosen to induce OS.

Despite the accumulating data in many species, such as human, ram and bovine [22,24,26,30,31,37,76], the role of melatonin in sperm physiology remains controversial. Regarding bovine spermatozoa, few studies have focused on the antioxidant properties of melatonin [26,29,35,77]. There is a wide range in the concentrations of melatonin used in these experiments, from nanomolar to millimolar, that could correspond to the different physiological concentrations of melatonin related to sex (male or female genital tract), different species [78] or experimental conditions [29].

In the present study, melatonin was supplemented in the medium at a 100 μM concentration, at which the molecule mediates an antioxidant role without damaging the membranes of spermatozoa [22,32,75]. The spermatozoa that were incubated for 60 min at 37 °C in the presence of 100 μΜ melatonin were more motile than the corresponding Control-t60 group, as determined by different motility parameters (Figure 2 and Figure 3, Table 1). In addition, melatonin inhibited or delayed cell death during the 60 min period as attested by the higher percentage of live spermatozoa at the end of the incubation (Figure 4). Melatonin not only retained the motility and viability of spermatozoa, but it also rendered them more capable in sustaining the insult by hydrogen peroxide in terms of motility (Table 1, Figure 3) compared to the Control-t60 group. The data comply with the results published by Li et al., which established that experimental conditions have a great impact on the outcome of melatonin supplementation [26]. Fernandez-Alegre et al. reported that melatonin differentially affects spermatozoa depending on the species and the experimental conditions [29]. In red deer, 0.1 mM or 1 mM melatonin did not improve sperm motility after a 4 h incubation [23]. On the other hand, the same concentration of melatonin (1 mM) improved the motility of human spermatozoa after a 30 min incubation [77] and the fertilizing capacity of normozoospermic and oligozoospermic samples [21].

The ameliorative effect of melatonin on bovine spermatozoa appears to be attributed to the antioxidant properties of melatonin. Relatively low concentrations of melatonin (10 μM) decreased the levels of ROS and improved the antioxidant status of sex-sorted bovine spermatozoa by increasing the activities of key antioxidant enzymes, such as glutathione peroxidase, superoxide ion dismutase and catalase [26]. Moreover, in the current study, the ameliorative effect of melatonin on the motility and viability of spermatozoa was accompanied by a concomitant increase in intracellular GSH levels (Figure 6), although it had no effect on superoxide ion levels (Figure 7). This could be attributed to the reaction of superoxide ions with NO to form peroxinitrate ions (ONOO^-^) [79], an interaction of highly reactive molecules characterized by Korkmaz et al. as the “devil’s triangle” [80]. Melatonin is known to directly scavenge ROS and NO and directly detoxify ONOO^-^ [81], thus reducing the reactive molecules in the cell milieu. On the other hand, melatonin may inhibit the production of NO from L-arginine. The enzymes that regulate this reaction are called NO synthases (NOS), and one particular isoform, the inducible NOS (iNOS), is regulated at the transcriptional level [82]. Therefore, we assessed the modulation of iNOS protein levels in bovine spermatozoa by melatonin to demonstrate that melatonin decreases the expression of iNOS in cells exposed to hydrogen peroxide (Figure 8). This is in agreement with the work of Gilad et al., who demonstrated that melatonin, at concentrations between 1 μM and 1 mM, inhibits the expression of iNOS in cultured murine macrophages stimulated with lipopolysaccharide [39]. Thus, melatonin may reduce not only ROS but also reactive nitrogen species (RNS) in spermatozoa. Alternatively, the beneficial effect of melatonin on the motility of spermatozoa could be attributed to changes induced in cytoskeletal elements of spermatozoa mediated by the interaction of melatonin with calmodulin, and preservation of ATP reserves, which are required to maintain flagellum activity [83]. Korkmaz et al. have stated that melatonin is the only currently available molecule which is known to block all aspects of the “devil’s triangle” as well as modulate the expression of antioxidant proteins, thereby preventing a redox imbalance and thus oxidative stress [80].

Nevertheless, the differential effects of melatonin on different types of cells appear to be cell-type- and concentration-dependent. As mentioned above, melatonin at micromolar concentrations modulates sperm capacitation in spermatozoa, whereas at nano- or picomolar concentrations it does not affect sperm motility [37]. These reports, along with the data presented here, clearly impose the need for further studies where a broad range of melatonin concentrations under different conditions (capacitating or not) will be tested in order to elucidate the mechanisms that mediate the effects of melatonin on spermatozoa.

## 5. Conclusions

The present study demonstrates that supplementation of media with melatonin reinforces the antioxidant status of spermatozoa and thus facilitates their endurance during thawing and handling in ART. In the presence of melatonin, spermatozoa retained their motility and viability at the end of experimentation, even under induced OS (exposure of spermatozoa to hydrogen peroxide). Melatonin elicits this effect either through its antioxidant activity or by inhibiting iNOS. We anticipate that these results will set a starting point for future studies in order to optimize the applications of melatonin in ART.

## Figures and Tables

**Table 1:** Progressive motility (PM) and kinematic parameters of spermatozoa as determined by CASA at the beginning (t0) and after 60 min of incubation at 37 °C (t60) in the presence or absence of 100 μM melatonin and 100 μM H_2_O_2_ (HP). The data are presented as mean ± SEM (*n* = 6). Different letters indicate statistically different values (*p* < 0.05).

Group	PM	VCL	VSL	VAP	ALH
Control-t0	21.16 ± 1.74	60.89 ± 3.17 ^a^	19.43 ± 1.19 ^a,b^	34.52 ± 1.87 ^a,b^	3.26 ± 0.16 ^a^
Melatonin-t0	23.77 ± 1.55	59.58 ± 1.22 ^a^	20.93 ± 0.5 ^a,b^	35.83 ± 0.46 ^a,b^	3.09 ± 0.08 ^a^
Control-t60	23.58 ± 2.23	49.82 ± 1.68 ^b^	18.55 ± 0.96 ^a,b^	30.47 ± 1.09 ^a,c^	2.72 ± 0.2 ^b,c^
HP	20.25 ± 4.62	43.72 ± 5.6 ^b^	17.83 ± 1.89 ^a^	23.85 ± 4.17 ^c^	2.93 ± 0.05 ^c^
Melatonin-t60	24.3 ± 4.38	62.15 ± 1.84 ^a^	21.55 ± 1.95 ^b^	36.67 ± 1.49 ^b^	3.38 ± 0.18 ^a^
Melatonin-HP	19.12 ± 2.81	54.38 ± 4.31 ^a,b^	18.4 ± 1.08 ^a,b^	32 ± 1.93 ^a,b^	3.18 ± 0.19 ^a,b,c^

PM: progressive motility, VCL: curvilinear velocity, VSL: straight-line velocity, VAP: average path velocity, ALH: amplitude lateral head movement.

## Data Availability

The data presented in this study are available upon request (S.N.L., slavrent@vet.auth.gr).

## Supplementary Materials

The following supporting information can be downloaded at: https://www.mdpi.com/article/10.3390/ani13203219/s1, Figure S1: original western blot figures for Figure 8B.
