# Supplementary figures and images for: Melatonin Protects Bovine Spermatozoa by Reinforcing Their Antioxidant Defenses
Source: Animals (Basel). 2023 Oct 15;13(20):3219. doi: 10.3390/ani13203219 (PMC10603642; doi:10.3390/ani13203219)

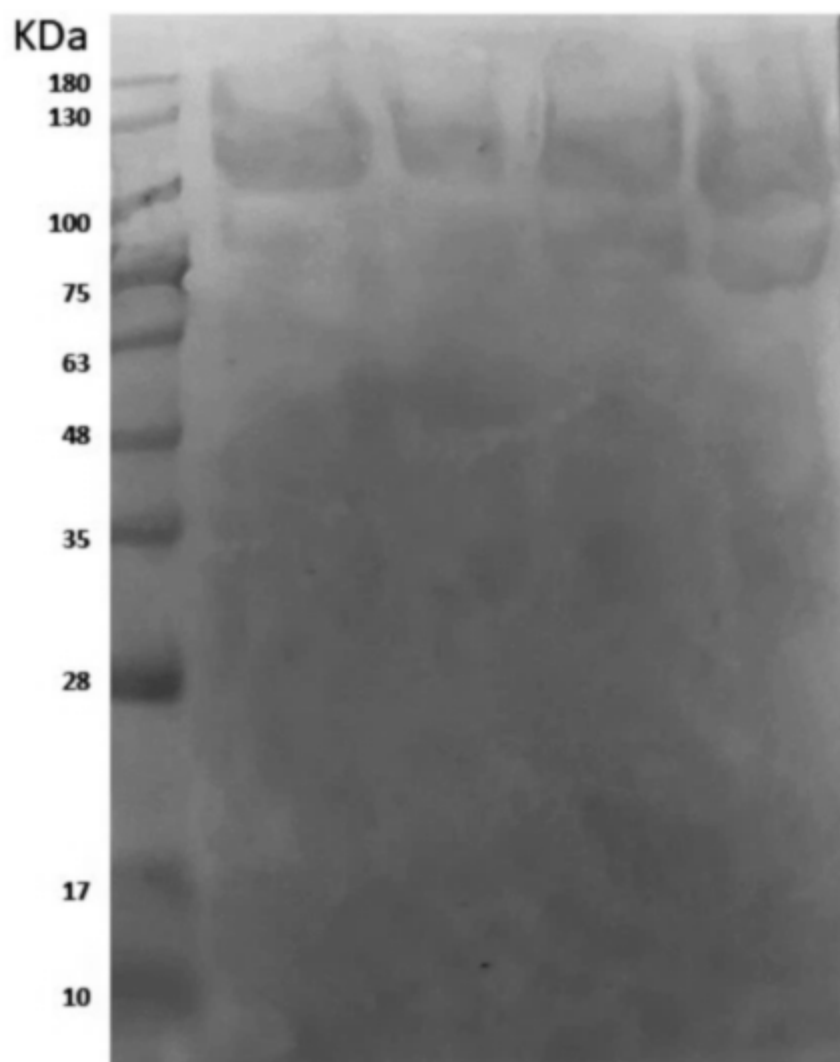

**Figure S1:** original western blot figures for Figure 8B.

Supplement: Supplementary file 1 [file animals-13-03219-s001.zip › animals-2614292-supplementary.pdf]
